# Influence of temperature on prevalence of health and welfare conditions in pigs: time-series analysis of pig abattoir inspection data in England and Wales

**DOI:** 10.1017/S0950268819002085

**Published:** 2020-02-18

**Authors:** H. Lee, C. Perkins, H. Gray, S. Hajat, M. Friel, R. P. Smith, S. Williamson, P. Edwards, L. M. Collins

**Affiliations:** 1 London School of Hygiene & Tropical Medicine, London, UK; 2 Faculty of Biological Sciences, University of Leeds, Leeds, UK; 3 Centre for Climate Change and Planetary Health, London, UK; 4 Animal and Plant Health Agency, Weybridge, Surrey, UK; 5 Animal and Plant Health Agency, Bury St Edmunds, Suffolk, UK

**Keywords:** Climate, epidemiology, estimating, impact of, prevalence of disease, respiratory infections, veterinary epidemiology

## Abstract

The prevalence of many diseases in pigs displays seasonal distributions. Despite growing concerns about the impacts of climate change, we do not yet have a good understanding of the role that weather factors play in explaining such seasonal patterns. In this study, national and county-level aggregated abattoir inspection data were assessed for England and Wales during 2010–2015. Seasonally-adjusted relationships were characterised between weekly ambient maximum temperature and the prevalence of both respiratory conditions and tail biting detected at slaughter. The prevalence of respiratory conditions showed cyclical annual patterns with peaks in the summer months and troughs in the winter months each year. However, there were no obvious associations with either high or low temperatures. The prevalence of tail biting generally increased as temperatures decreased, but associations were not supported by statistical evidence: across all counties there was a relative risk of 1.028 (95% CI 0.776–1.363) for every 1 °C fall in temperature. Whilst the seasonal patterns observed in this study are similar to those reported in previous studies, the lack of statistical evidence for an explicit association with ambient temperature may possibly be explained by the lack of information on date of disease onset. There is also the possibility that other time-varying factors not investigated here may be driving some of the seasonal patterns.

## Introduction

### Background

Climate change is leading to fundamental changes in weather patterns which have the potential to impact greatly on human and animal health [1, 2]. The issue of climate change and agriculture is high on the global health agenda, with sustainable intensification of agriculture suggested as a potential solution to increasing food production whilst mitigating negative environmental impacts [3]. For livestock production, animal health and welfare is an important contributing factor for sustainable agriculture [4], as healthy animals are more efficient and require less medication. Although the links between climate factors and selected infectious diseases in humans are becoming increasingly well characterised [5], the role of climate in animal health and welfare is less clear [6]. Increasing our understanding of the links between climate and disease will enable better control and alleviation of disease burdens in livestock farming and contribute to the future sustainability of the sector.

Climate change can have direct and indirect impacts on the health and welfare of livestock. Direct effects may be caused by the action of increased temperature leading to increased metabolic disorders and mortality [7]. Indirect effects come from many sources, including altered distributions of vector species, changes in the biology of the pathogens and/or the establishment of new microenvironments [2, 8].

In intensive pig production, considerable efforts are made to control the environment in order to optimise health and production performance [9]. In spite of this, seasonal patterns are reported for several diseases, including Porcine Reproductive and Respiratory Syndrome, *Klebsiella septicaemia,* enzootic pneumonia (EP) and respiratory conditions [10–14], indicating a potential role for climate factors. Seasonal variations in non-respiratory conditions (e.g. pericarditis and tail damage) have also been reported at slaughter in the UK [15]. Few studies have tried to further identify associations between weather factors and patterns of health and welfare conditions. For example, McCormick *et al*., identified relationships between climate factors and disease, showing papular dermatitis, EP and milk spots were associated with temperature [16]. However, significant gaps remain in our understanding of the underlying drivers of these observed temporal patterns, and the extent to which they can be explained by ambient temperature and other climatic factors.

Respiratory conditions and tail biting cause poor health and welfare states and have been associated with poor performance, increased production costs [17] and economic loss [18]. Respiratory conditions can reduce pig growth, increase feed conversion ratios [19] and may result in additional medication costs. Factors associated with the onset of respiratory conditions include stocking density, control of climatic conditions (e.g. ventilation, temperature control) and biosecurity measures [20, 21]. Another significant issue for pig production is the prevalence and severity of tail biting, which is often used as an indicator of pig welfare [11, 22]. There are multiple proposed risk factors for tail biting including: feeding practices, enrichment provision and ventilation [22, 23], as well as thermal factors including draughts, temperature variation, chilling and over-heating [24–26].

### Objectives

Using abattoir-collected data and by applying time-series regression methods, this study characterises the relationship between ambient temperature and the prevalence of respiratory conditions and tail biting in pigs in England and Wales.

## Methods

### Datasets

#### Abattoir inspection data

All pigs slaughtered for human consumption in England and Wales are audited and controlled by the Food Standards Agency. Statutory pig health and welfare data (Collection and Communication of Inspection Results – CCIR) collected at slaughter was the animal health and welfare data source. The proportion of conditions present in the pigs slaughtered at abattoirs was identified as a reasonable proxy for prevalence in the field, as almost all pigs produced in the UK are slaughtered in UK abattoirs, with very few live animals imported and even fewer exported. Pig movement data from the Scottish livestock traceability research team and an electronic licence for pig movements shows that 0.3% of pigs sent to slaughter in Great Britain in 2016–2017 were born or reared in a non-UK country, and 97.7% of these were from Ireland.

Pigs arrive at the abattoirs in batches, defined as a group of pigs from the same herd and farm of origin delivered to the abattoir on the same day. Ante- and post-mortem inspections are carried out at all abattoirs by official auxiliaries (commonly known as meat hygiene inspectors (MHIs)) and veterinarians. Conditions detected in each pig during the inspections are recorded in the CCIR at the batch level. Information in the dataset includes: (i) date of slaughter; (ii) batch identification number; (iii) total number of animals in the batch; (iv) description of conditions; (v) condition counts; (vi) body part where the condition occurs; (vii) inspection type and (viii) producer postcode. The CCIR datasets were obtained for the period from January 2010 to December 2015 and used to assess prevalence of respiratory and tail biting conditions at national (England and Wales) and county levels. County level analysis was chosen as the highest spatial resolution possible due to the confidentiality agreements with data providers.

To assess county-level prevalence, the CCIR records for England and Wales were assigned to counties using farm postcodes. Counties were assigned based on the UK Ceremonial County boundaries as defined by the *Lieutenancies Act 1997* [27]. Where CCIR postcodes were incomplete or included recording errors, the first one or two letter(s) of the postcode, or the city name, were used to link the records to postcode areas using the Office for National Statistics (ONS) Postcode Directory User Guide (February 2018, ONS Geography).

Of a total 4 916 898 records, 4 905 910 had geographic information assigned to them in the CCIR. Of these, 105 000 (2.14%) could not be matched to a county: 15 295 (0.31%) had missing values in postcodes and 89 705 (1.82%) had incomplete postcodes or invalid values (further information available in Supplementary Figure S1: Data cleaning process).

#### Weather data

The daily maximum temperature was sourced from the Met Office National Climate Information Centre (NCIC). For national level analyses, temperatures were obtained from the Central England Temperature (CET) series, which is representative of a roughly triangular area of the United Kingdom enclosed by Lancashire, London and Bristol [28]. This was the best available representation of a national level temperature time series. Correlations between daily maximum, minimum and mean temperatures were very high, but maximum values are considered here to also capture extreme heat periods.

To generate county level temperature datasets, all NCIC stations were assigned to counties and a composite series for each county was calculated as the average of all stations within each county. Of 56 counties in England and Wales, 53 had complete temperature data: three had insufficient data (city and county of the city of London; Mid Glamorgan and Tyne and Wear).

### Data analyses

CCIR data were initially collapsed by date of slaughter to create a daily time series. To account for the day of week effect (influenced by abattoirs' operational days) all data were combined into weekly series and analyses conducted at the weekly level.

#### Prevalence of respiratory conditions and tail biting

All records from the CCIR for respiratory conditions and tail biting were included regardless of the inspection types (i.e. ante- and post-mortem inspections). The following conditions from the CCIR were aggregated to generate one category of respiratory conditions: Abnormal breathing rate/depth; Abnormal respiratory signs; Coughing; Respiratory; Rhinitis; Pneumonia; Pleurisy; Pericarditis; OR Twisted snout. Conditions recorded as tail biting were categorised as tail biting.

Using the unique batch identification numbers and date of slaughter, weekly time-series datasets were created for respiratory and tail biting prevalence. For all of the selected respiratory conditions, the daily number of positive results in each week was summed. The weekly totals were then divided by the total number of pigs slaughtered in the same week (using batch ID, number of pigs in batch and date of slaughter) to generate prevalence at national and county levels. The prevalence of tail biting was calculated using the same method. The data cleaning and aggregation process are summarised in Supplementary material (Supplementary Figure S1: Data cleaning process).

#### Statistical methods

Time-series regression analysis was used to assess the relationships between weekly prevalence of the outcomes and weekly maximum ambient temperature. Spline functions were used to control for broad seasonal patterns and long-term trends in the data. The maximum temperature was lagged by up to 10 weeks from the date of slaughter to capture delayed effects of exposure. Distributed lag models were used to simultaneously model the separate effects of each weekly lag after adjustment for all other lags.

The most appropriate lag was chosen based on the comparison of model deviances. Natural cubic splines were used to determine the functional form of the relationship between temperature and disease prevalence and possible temperature threshold values, at which the relationship with prevalence is seen to change. To allow comparison of effect estimates between counties, optimum thresholds were determined based on fitting all possible temperature values between the 1st and the 99th percentile of the temperature distribution within each county, and then the percentile value with the lowest summed deviance across counties was used as the threshold in each county-level model. Results are presented as a relative risk (RR) per 1 °C increase (or decrease) in temperature above (or below) thresholds. A random effects meta-analysis of county-level estimates was conducted to obtain a pooled effect. Similar analyses were conducted for tail biting as the outcome measure. Regression analyses were performed using Stata v.15.1 (StataCorp LP, College Station, Texas) and geographical analyses using ArcGIS 10.5 (Esri UK).

After the initial analysis of the CCIR dataset, it was found that in some instances the total number of pigs slaughtered per year exceeded independently reported national production numbers (particularly for the year 2014). After recognition of these inconsistencies, sensitivity analyses were conducted on national and county models in order to determine how removal of such outliers impacted modelled outcomes.

## Results

### Descriptive data

The CCIR data in the national level dataset collected from January 2010 to December 2015 is summarised in Table 1. The prevalence of respiratory conditions was much greater than tail biting, with a weekly average of nearly 20% compared to less than 1% for tail biting, at the national level. Of the total respiratory conditions recorded, pleurisy, pericarditis and pneumonia accounted for the majority (over 98%).

**Table 1.** Summary of the CCIR dataset from January 2010 to December 2015 (*n* = 4 916 898; no missing data)

	Mean	Standard deviation	Minimum, maximum
Prevalence of respiratory conditions per week	19.55%	1.60	15.55, 24.61
Total number of cases of respiratory conditions per week	28 823.80	19 432.27	10 819, 234 389
Prevalence of tail biting per week	0.65%	0.20	0.24, 1.36
The total number of cases of tail biting per week	987.96	872.83	232, 10920

Pigs originating from five counties (North Yorkshire, East Riding of Yorkshire, Norfolk, Suffolk and Lincolnshire) accounted for a large proportion (65.95%) of the total. There were 11 counties (East Sussex, Merseyside, Surrey, West Glamorgan, South Glamorgan, Bristol, Rutland, Greater London, Mid Glamorgan, Tyne and Wear and Isle of Wight) that contributed fewer than 4000 records (0.08%) each throughout the study period.

Based on the CET dataset, the maximum ambient temperature averaged 13.82 °C (s.d. 5.56; min −1.09 and max 26.31).

### Seasonal patterns of prevalence

**Fig. 1.**
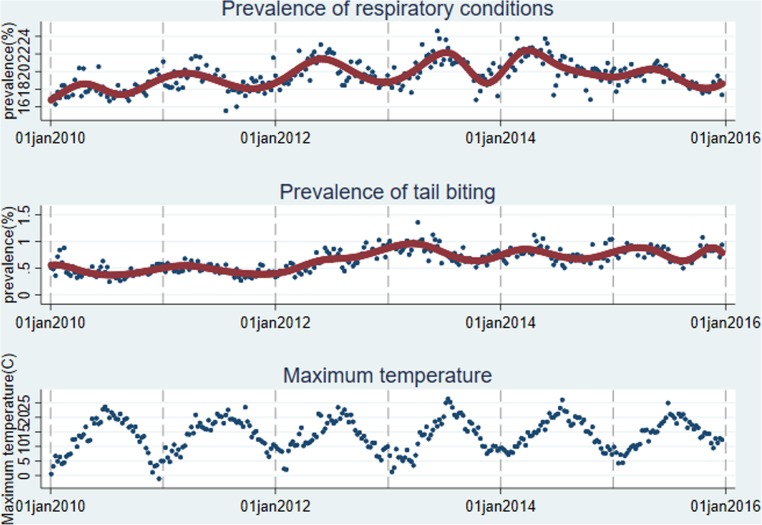
Weekly prevalence of respiratory and tail biting conditions in the CCIR dataset at the national level and maximum ambient temperature from January 2010 to December 2015.

The national-level prevalence of the selected conditions is shown in Figure 1. There were annual peaks and troughs in prevalence of respiratory conditions, with peaks often occurring during the first 6 months of the year and troughs in the fourth quarter (October to December). The prevalence of tail biting from January 2010 to December 2015 ranged between 4% and 10%, and below 5% in most counties during the study period (further illustrated in Supplementary Figure S2).

At the county level, all counties showed cyclic patterns in prevalence of respiratory conditions throughout the study period, whilst the difference between seasonal peaks and troughs in prevalence varied between 20% and 80% (further detailed in Supplementary Figure S3).

### Prevalence of conditions and ambient temperature

#### Respiratory conditions

**Fig. 2.**
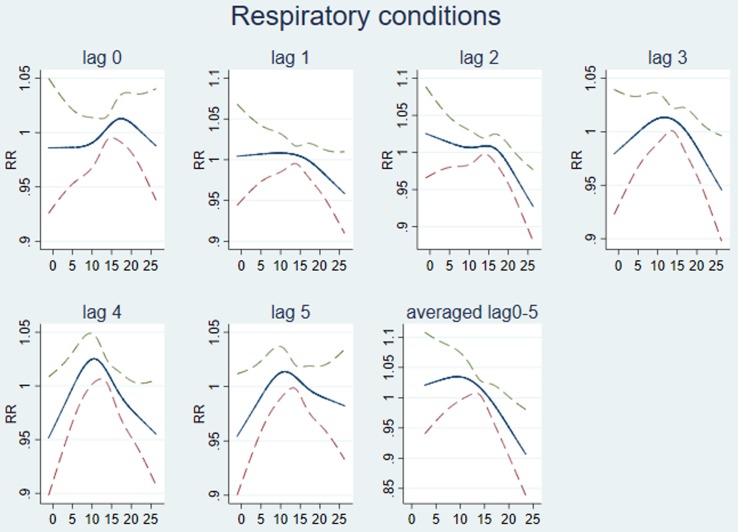
Modelling lagged association between maximum temperature and respiratory conditions. Graphs lag 0 to lag 5 show a single lag at a time (the unit of lag is weeks). The last graph (averaged lag 0–5) is fitted lags from week 0 to week 5 together. RR (95% CI) of respiratory conditions (*y*-axis) by maximum temperature (°C; *x*-axis).

The association between temperature and respiratory conditions was explored using lags of between 0 and 10 weeks. The best fitting exploratory national models used temperature lags of between 0 and 5 weeks (Fig. 2). For the national datasets, RR of respiratory conditions peaks when the maximum temperature reaches ~15 °C and the prevalence decreases linearly below and above this threshold (Fig. 2). In the final distributed lag 0–5 model, we estimated a national RR of 1.006 (95% CI 0.823–1.229; *P*-value 0.956) per one-degree decrease in temperature below 15 °C and a RR of 0.991 (95% CI 0.789–1.244; *P*-value 0.937) per one-degree increase in temperature above this threshold.

**Fig. 3.**
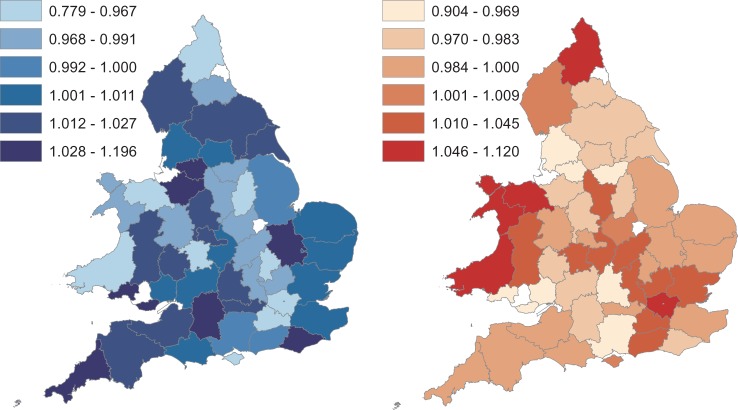
Seasonally-adjusted relationship between temperature (using the distributed lag model between 0–5 weeks) and prevalence of respiratory disease in 50 counties in England and Wales, January 2010 to December 2015 (except Dyfed from January 2011 to December 2015): RR of respiratory conditions per one-degree decrease below cold thresholds (left) and one-degree increase above heat thresholds (right). White areas are excluded counties (Mid Glamorgan, Tyne & Wear, Bristol, Rutland and Merseyside).

At the county level, the optimum temperature thresholds for both cold and heat effects were determined to be at the 46th percentile of lag 0 temperature. The 46th percentile temperature ranged between 8.2 and 14.6 °C across the counties (and equates to 13.5 °C in national data from the CET). The effects on respiratory morbidity of one-degree changes above and below this maximum threshold temperature in each county were mixed and are shown in Figure 3. Counties in Wales, along with Greater London and Northumberland showed the highest increases in respiratory conditions with an increase in temperatures, while Counties in the South West, East of England and the North showed the highest increases in respiratory conditions with a decrease in temperature below the threshold. In many counties a decrease in RR as temperature increased over thresholds was seen. The pooled effect per one-degree decrease in temperature below the threshold temperature for each county using the distributed lag 0–5 model was a small increase in the RR of 1.004 (95% CI 0.969–1.039). The overall effect per one degree increase above the same threshold was a decrease in the RR of 0.992 (95% CI 0.9582–1.026).

#### Tail biting

**Fig. 4.**
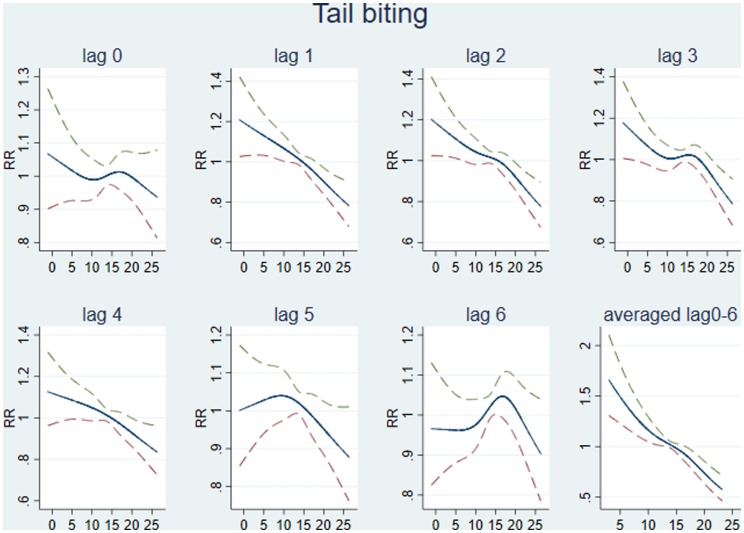
Modelling lagged association between maximum temperature and tail biting. Graphs from lag 0 to lag 6 have been fitted with a single lag at a time. The last graph (averaged lag 0–6) is fitted lags from week 0 to week 6 together. The unit of lag is in weeks. RR (95% CI) of tail biting conditions (*y*-axis) by maximum temperature (°C; *x*-axis).

The association between temperature and tail biting was explored using lags of between 0 and 10 weeks. At the national level, across most lags there was an increased prevalence in tail biting associated with a reduction in temperature across the full range of the temperature scale (Fig. 4). The best fitting national model was a distributed lag model across weeks 0–6, which resulted in a slightly increased RR of 1.053 (95% CI 0.189–5.882; *P*-value 0.953) for every one degree drop in temperature (also see Supplementary Table S1: Sensitivity analysis).

**Fig. 5.**
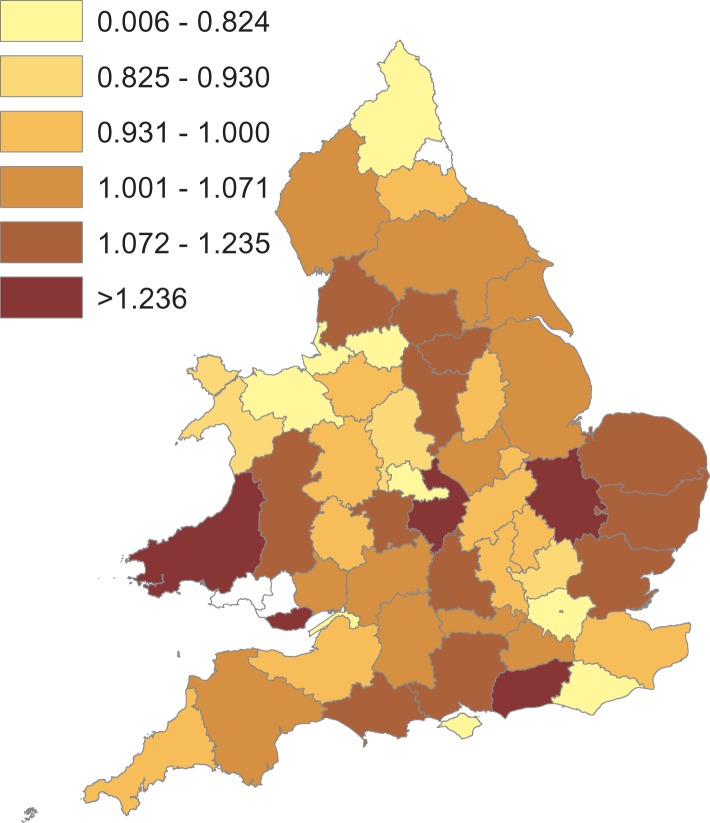
RR of tail biting conditions with one-degree decrease after adjusting for seasonality (using distributed lag between 0–6 weeks) and prevalence of tail biting in 52 counties in England and Wales, January 2010 to December 2015 (except Dyfed from January 2011 to December 2015): White is excluded counties (Mid Glamorgan, West Glamorgan and Tyne & Wear).

Distributed lag models across weeks 0–6 developed at the county level showed varying results across counties. The highest increases in RR of tail biting associated with a drop in temperature were found in the south of Wales, Warwickshire, Cambridgeshire and West Sussex, with the majority of counties showing a small increase in RR (Fig. 5). The overall pooled effect for all counties was a slight increase in the RR of 1.028 per one-degree decrease in temperature (95% CI 0.776–1.363).

Sensitivity analyses conducted for respiratory conditions and tail biting at the national and county levels found that model outputs remained consistent when anomalous CCIR data were excluded using different methods (Supplementary Table S4: Sensitivity analysis for national models and Supplementary Table S5: Sensitivity analysis for county models).

## Discussion

We found seasonal changes in the prevalence of respiratory conditions and of tail biting at the national level in pigs slaughtered in England and Wales between 2010 and 2015. However, there was no statistical evidence of an effect of the maximum temperature on respiratory conditions or tail biting at the national or county levels. Any changes in RR were small in magnitude and high in uncertainty.

### Previous studies

We found seasonal changes in the prevalence of respiratory conditions and of tail biting at the national level which is in agreement with previous analyses [10, 11, 15, 22, 26, 29, 30]. The association between ambient temperature and mortality in both humans and animals has been reported to fit a J- or U-shaped curve, with extreme temperatures being linked with increased mortality [31–35] and respiratory morbidity [36]. In this study, the relationship with outcomes was better described by an A-shaped curve in the national dataset, with inconsistent patterns displayed across the county-level datasets. The lack of a clear association found in this study may be the result of a number of factors that impact the prevalence of conditions in the CCIR which are not included in the model. We hypothesise that seasonal trends in the occurrence of these conditions could be related to management practices and other factors, such as housing, ventilation and pathogen presence, rather than weather conditions *per se*, or it may reflect multifactorial outcomes that are too complex to model using the techniques employed here.

There may also be additional weather factors that are related to the conditions. For instance, studies on respiratory virus transmission in humans have shown that different environmental factors (including humidity, precipitation and air flow) can be determinants of infection and transmission [37]. Paynter *et al*., [38] reported an increase in virus transmission when conditions were cold and dry. One study of Belgian farms found that as indoor temperature decreased, the occurrence of coughing among pigs increased [25].

The current study found a seasonal pattern in tail biting at the national level, with the pattern of tail biting increasing as temperature decreased, although this effect was uncertain. Seasonal trends in tail biting have been reported in other studies [22] including increases of occurrence in colder months [39, 40], with large fluctuations in temperature during the day and night also being implicated in outbreaks [41]. Studies have associated tail biting with thermal stress; with a higher risk of episodes when temperatures are either above or below the thermal comfort zone for pigs [22]. In the present study, the overall prevalence of tail biting was typically lower than 1%. Similarly, Harley *et al*., [42] found that the prevalence of severe tail lesions scored in abattoirs in Northern Ireland and the Republic of Ireland was 1.03%, whilst the prevalence of mild tail lesions was 58.1%. Whilst scoring of the severity of lesions is not conducted in the routine meat inspections that comprise the CCIR data, it seems plausible that the tail lesions being recorded were of a severe nature. The lack of strong seasonal variation in tail biting reported in the current study may be due to ventilation and housing protecting pigs from reaching the level of thermal stress required to affect tail biting prevalence in different seasons and the under reporting of less severe cases/lesions. The lack of strong association with ambient maximum temperature may suggest there are other factors affecting tail biting. Short-term temperature fluctuations, increased draughts and extreme temperatures may all be implicated as contributors to the seasonal trend in tail biting [26, 43]. As tail docking use was not recorded as part of this dataset, it is not possible to ascertain the impact of docking on the relationship between tail biting and climate factors.

### Strengths and weaknesses of the study

Unlike conventional applications of advanced time-series regression methods developed and widely used in the human environmental epidemiology field, this study applied the methods to an extensive animal health dataset. The current study benefits from the use of the high coverage CCIR dataset, with 6 years of data, allowing trend analysis and good specificity in identifying the conditions [44]. As the scheme represents statutory reporting, all pigs sent to abattoirs in England and Wales were assessed and included in this dataset [44]. The current study has a large sample size which increased the precision of the study. Additionally, the 6-year period allows us to draw reliable results regarding seasonal changes in the prevalence of respiratory conditions and tail biting over this time period.

There are a number of limitations to the current study. Firstly, there are those arising from the use of the CCIR dataset. The CCIR only records the presence of conditions on the date of slaughter and contains no information on condition onset. The use of lag models addressed this issue to some extent, however, it remains the case that exposures can only be linked to the date of slaughter and not to disease onset. This may lead to an under reporting of conditions if mortality or culling occurred on farms [41]. Conditions in the CCIR dataset are recorded at the batch level rather than for individual pigs. Where an individual pig displayed several conditions this resulted in one record for each observed condition. Aggregating respiratory conditions may have resulted in some double counting of pigs which may have masked patterns and associations at the individual condition level and caused a reduced sensitivity to detect associations. CCIR data only contains information on pigs deemed fit enough to send to slaughter, with no data available on mortality and culls that occur on farm. Information regarding the farm system and management, which might be related to the disease prevalence (e.g. level of biosecurity), is not available. There is evidence that the CCIR data is accurate at a national level but less so at the batch level, with a number of issues potentially impacting its quality and completeness, including lack of routine data cleaning. We conducted sensitivity analyses at the national level to explore some of the issues highlighted in the data cleaning process (available in Supplementary Table S4) and found only small variations in the model output. Only minimal evaluations are carried out on MHIs, meaning that less severe or obvious conditions may be under reported and the sensitivity of detection may vary [11, 44]. Secondly, in this current study, there may have been some misclassification of postcode information in the data cleaning process. Records were assigned to counties based on the postcode in the CCIR data and some misclassification bias may have been introduced by using the initial letter(s) of a postcode or the city name to assign records to counties. However, these records comprised only 5.51% (259 141 out of 4 704 156) of the total records in the dataset before aggregating to weekly level. Finally there are large differences in pig density across the study area [45] and pooled effect estimates across counties were weighted according to standard errors rather than how many pigs each county contributed. This was selected as the best available method as it gave greater weight to the county models with the statistically strongest estimates.

### Implications and future research

We need to be cautious when interpreting these results across different contexts because the data were from pigs slaughtered at abattoirs in England and Wales only, with limited information on other risk factors. Results differed by county, which could reflect a number of systems-based variations in the different areas such as farm type, breed and whether farm practices consistently differed in different areas.

The comparison in this study of aggregated ambient temperature with conditions at the county and national levels may have resulted in the masking of more localised patterns. Further investigations would benefit from localised weather data and temperature in the pig enclosures. With further information about the temperature at the farm level, alongside more detailed information on morbidity and mortality, we may be able to understand the relationship between environmental factors and the prevalence of different health and welfare conditions in livestock. We hypothesise that the lack of statistical evidence of an association between ambient temperature and conditions in the CCIR data could indicate a true absence of association, may have been contributed to by missing information on disease onset, or the relationship may be much more complicated than simple ambient temperature and other factors, i.e. temperature fluctuations (either long term or short term) may be more important. In the latter scenario, an increase in these fluctuations that could be caused by climate change could be a problem for the future pig health and welfare. Further investigations using a more standardised method of abattoir data collection, with high geographical and temporal coverage and various weather factors could clarify this and provide information on the potential for these datasets to provide insights into the relationship between climate and pig health and welfare.

## Conclusion

We found seasonal variation in the prevalence of respiratory conditions and of tail biting at the national level. However, there was no statistical evidence of an association between the maximum temperature and the prevalence of respiratory conditions or tail biting in pigs. The prevalence of these conditions could be related to factors other than ambient temperature, or it may reflect factors too complex to model using time-series regression models. The lack of information on disease onset may also have contributed to the lack of evidence of an association. Further investigations of abattoir data combined with additional localised weather, farm level information and pig health data could reveal further insights into the factors affecting respiratory conditions and tail biting in pigs.

## Supplementary material

For supplementary material accompanying this paper visit https://doi.org/10.1017/S0950268819002085. click here to view supplementary material

## PigSustain project

The PigSustain aims to use a multi-disciplinary, integrated systems approach to model and assess the resilience of the UK pig industry historically, currently and in the future. In this study, we investigated the association between climate change and health. As a larger piece of the work, this study will contribute to developing models to assess how the industry will likely be affected by intensification, fluctuations in consumer demand, climate change and risks associated with global production and international trade.
