# Supplementary material for: Influence of temperature on prevalence of health and welfare conditions in pigs: time-series analysis of pig abattoir inspection data in England and Wales
Source: Epidemiol Infect. 2020 Feb 18;148:e30. doi: 10.1017/S0950268819002085 (PMC7026902; doi:10.1017/S0950268819002085)
Supplement: Supplementary file 1 [file S0950268819002085sup001.zip › S0950268819002085sup001/Supplementary Table S4 Sensitivity analysis national.docx]

Supplementary Table S4: Sensitivity analysis for national models

The number of pigs in the CCIR dataset for 2014 was found to exceed independently reported national production numbers. Sensitivity analyses were conducted on national and county models in order to determine how the removal of such outliers impacted modelled outcomes.

| **Dataset** | **Respiratory conditions** | | **Tail biting** |
| --- | --- | --- | --- |
|  | RR per one-degree increase in temperature above threshold | RR per one-degree decrease in temperature below threshold | RR per one-degree decrease in temperature |
| Original reported | 0.991 (95% CI 0.789, 1.244; p-value 0.937) | 1.006 (95% CI 0.823, 1.229; p-value 0.956) | 1.053 (95% CI 0.189, 5.882; p-value 0.953) |
| Abattoirs removed for 2014* | 0.991 (95% CI 0.790, 1.243; p-value 0.937) | 1.005 (95% CI 0.823, 1.228; p-value 0.958) | 1.033 (95% CI 0.461, 2.312; p-value 0.938) |
| 13 weeks data removed** | 0.989 (95% CI 0.776, 1.261; p-value 0.931) | 1.006 (95% CI 0.822, 1.230; p-value 0.956) | 1.051 (95% CI 0.187, 5.897; p-value 0.955) |
| Pigs >10000 removed*** | 0.991 (95% CI 0.790, 1.242; p-value 0.934) | 1.006 (95% CI 0.824, 1.228; p-value 0.954) | 1.032 (95% CI 0.461, 2.309; p-value 0.939) |
| Pigs>8000 removed**** | 0.991 (95% CI 0.790, 1.242; p-value 0.936) | 1.006 (95% CI 0.824, 1.228; p-value 0.956) | 1.032 (95% CI 0.462, 2.307; p-value 0.939) |

*Three abattoirs were identified as visual outliers, slaughtering a significantly larger than expected number of batches of pigs per day within 2014, and were therefore removed entirely from the dataset for that year. This excluded the most data, while subsequent analyses investigated the effects of more targeted removal of anomalous data.

**Thirteen weeks were identified where weekly pig totals exceeded the maximum weekly slaughter capacity (set at 203,000 according to AHDB weekly clean pig slaughter statistics from 2014-2019), these weeks of data were removed from the analysis.

*** All data were omitted from the analysis from days in which more than 8000 pigs were slaughtered at an abattoir.

****All data were omitted from the analysis from days in which more than 10000 pigs were slaughtered at an abattoir.
