# Supplementary material for: Influence of temperature on prevalence of health and welfare conditions in pigs: time-series analysis of pig abattoir inspection data in England and Wales
Source: Epidemiol Infect. 2020 Feb 18;148:e30. doi: 10.1017/S0950268819002085 (PMC7026902; doi:10.1017/S0950268819002085)
Supplement: Supplementary file 1 [file S0950268819002085sup001.zip › S0950268819002085sup001/Supplementary Table S5 Sensitivity analysis county.docx]

Supplementary Table S5: Sensitivity analysis for county models, utilising data from the five counties which contributed the most pigs (East Riding of Yorkshire, Lincolnshire, Norfolk, North Yorkshire, Suffolk) and two of the counties that contributed some of the least (Merseyside, East Sussex).

| Dataset | County | **Respiratory conditions** | | **Tail biting** |
| --- | --- | --- | --- | --- |
|  |  | RR per one degree decrease in temperature below threshold (46^th^ percentile) | RR per one degree increase in temperature  above threshold (46th percentile) | RR for every one degree drop in temp |
|  |  | RR (95% CI; p-value) | RR (95% CI; p-value) | RR (95% CI; p-value) |
| Original | East Riding of Yorkshire | 1.023 (95% CI 0.820, 1.275; p value 0.843) | 0.973 (95% CI 0.773, 1.224; p-value 0.814) | 1.051 (95% CI 0.167, 6.634; p value 0.958) |
|  | Lincolnshire | 0.996 (95% CI 0.821, 1.208; p value 0.967) | 0.998 (95% CI 0.815, 1.223; p-value 0.986) | 1.065 (95% CI 0.229, 4.947; p value 0.936) |
|  | Norfolk | 1.008 (95% CI 0.778, 1.305; p value 0.954) | 0.996 (95% CI 0.770, 1.288; p-value 0.973) | 1.142 (95% CI 0.080, 16.390; p value 0.922) |
|  | North Yorkshire | 1.012 (95% CI 0.807, 1.268; p value 0.919) | 0.980 (95% CI 0.769, 1.249; p-value 0.869) | 1.055 (95% CI 0.194, 5.725; p value 0.950) |
|  | Suffolk | 1.005 (95% CI 0.811, 1.246; p value 0.961) | 0.997 (95% CI 0.801, 1.241; p-value 0.980) | 1.156 (95% CI 0.119, 11.253; p value 0.901) |
|  | Merseyside | 1.021 (95% CI 0.849, 1.229; p value 0.823) | 1.059 (95% CI 0.761, 1.474; p-value 0.732) | 0.502 (95% CI 0.027, 9.188; p value 0.643) |
|  | East Sussex | 1.078 (95% CI 0.794, 1.464; p value 0.63) | 0.970 (95% CI 0.735, 1.280; p-value 0.829) | 0.459 (95% CI 0.000, 481239; p value 0.912) |
| 8k* | East Riding of Yorkshire | 1.019 (95% CI 0.819, 1.268; p value 0.866) | 0.973 (95% CI 0.775, 1.221; p-value 0.814) | 1.046 (95% CI 0.166, 6.591; p value 0.962) |
|  | Lincolnshire | 1.000 (95% CI 0.826, 1.210; p value 0.997) | 0.995 (95% CI 0.814, 1.215; p-value 0.957) | 1.081 (95% CI 0.238, 4.905; p value 0.919) |
|  | Norfolk | 1.010 (95% CI 0.780, 1.307; p value 0.94) | 0.992 (95% CI 0.768, 1.280; p-value 0.948) | 1.184 (95% CI 0.088, 15.842; p value 0.898) |
|  | North Yorkshire | 1.011 (95% CI 0.810, 1.263; p value 0.922) | 0.982 (95% CI 0.771, 1.251; p-value 0.886) | 1.062 (95% CI 0.199, 5.672; p value 0.944) |
|  | Suffolk | 1.009 (95% CI 0.815, 1.249; p value 0.937) | 0.993 (95% CI 0.799, 1.233; p-value 0.948) | 1.137 (95% CI 0.122, 10.613; p value 0.910) |
|  | Merseyside | 1.026 (95% CI 0.853, 1.233; p value 0.788) | 1.055 (95% CI 0.759, 1.465; p-value 0.752) | 0.573 (95% CI 0.032, 10.139; p value 0.704) |
|  | East Sussex | 1.078 (95% CI 0.794, 1.464; p value 0.630) | 0.970 (95% CI 0.735, 1.280; p-value 0.829) | 0.554 (95% CI 0.000, 535700; p value 0.933) |
| 10k** | East Riding of Yorkshire | 1.022 (95% CI 0.820, 1.274; p value 0.847) | 0.973 (95% CI 0.775, 1.221; p-value 0.813) | 1.046 (95% CI 0.166, 6.601; p value 0.961) |
|  | Lincolnshire | 1.000 (95% CI 0.826, 1.211; p value 0.998) | 0.994 (95% CI 0.814, 1.214; p-value 0.952) | 1.080 (95% CI 0.238, 4.895; p value 0.921) |
|  | Norfolk | 1.011 (95% CI 0.781, 1.308; p value 0.935) | 0.991 (95% CI 0.768, 1.280; p-value 0.946) | 1.182 (95% CI 0.088, 15.804; p value 0.899) |
|  | North Yorkshire | 1.010 (95% CI 0.808, 1.263; p value 0.929) | 0.983 (95% CI 0.772, 1.252; p-value 0.888) | 1.064 (95% CI 0.199, 5.697; p value 0.942) |
|  | Suffolk | 1.009 (95% CI 0.815, 1.250; p value 0.932) | 0.992 (95% CI 0.799, 1.233; p-value 0.946) | 1.137 (95% CI 0.122, 10.615; p value 0.910) |
|  | Merseyside | 1.021 (95% CI 0.849, 1.229; p value 0.823) | 1.059 (95% CI 0.761, 1.474; p-value 0.732) | 0.573 (95% CI 0.032, 10.139; p value 0.704) |
|  | East Sussex | 1.078 (95% CI 0.794, 1.464; p value 0.63) | 0.970 (95% CI 0.735, 1.280; p-value 0.829) | 0.554 (95% CI 0.000, 535700; p value 0.933) |

* All data were omitted from the analysis from days in which more than 8000 pigs were slaughtered at an abattoir.

**All data were omitted from the analysis from days in which more than 10000 pigs were slaughtered at an abattoir.
