# Supplementary figures and images for: Influence of temperature on prevalence of health and welfare conditions in pigs: time-series analysis of pig abattoir inspection data in England and Wales
Source: Epidemiol Infect. 2020 Feb 18;148:e30. doi: 10.1017/S0950268819002085 (PMC7026902; doi:10.1017/S0950268819002085)

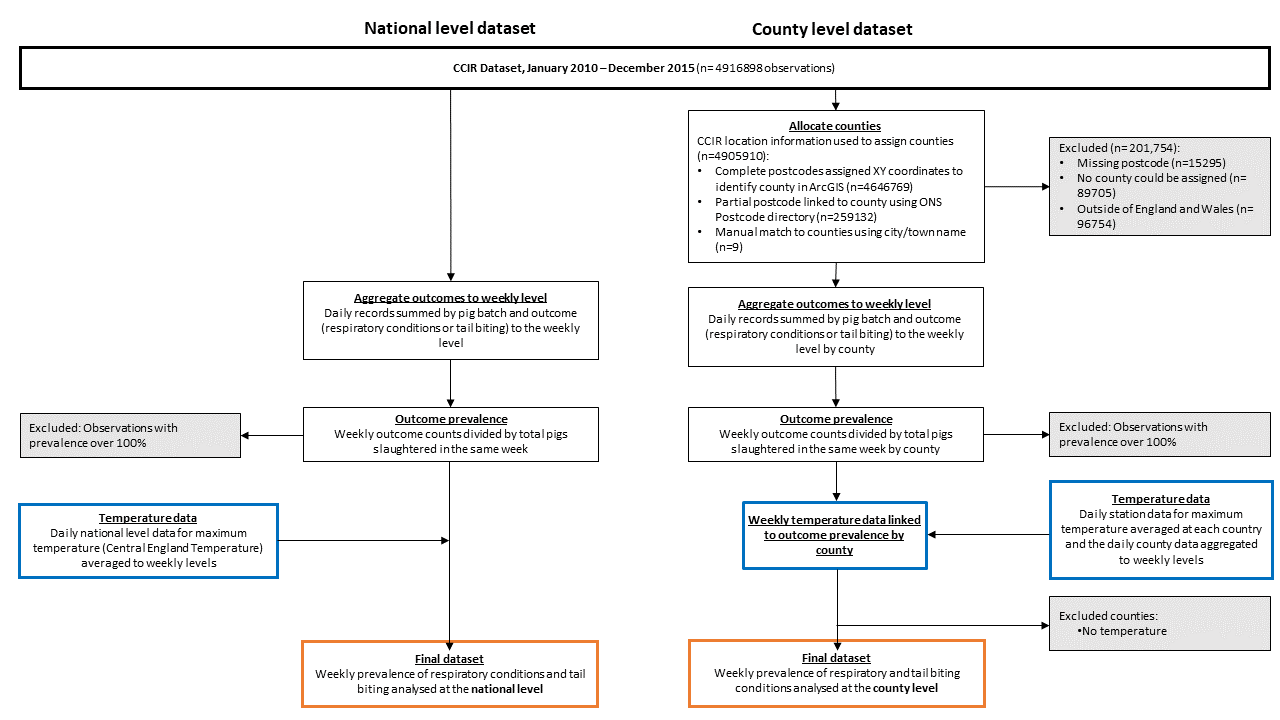

Supplement: Supplementary file 1 [file S0950268819002085sup001.zip › S0950268819002085sup001/Supplementary Figure S1 Data cleaning process.tif]

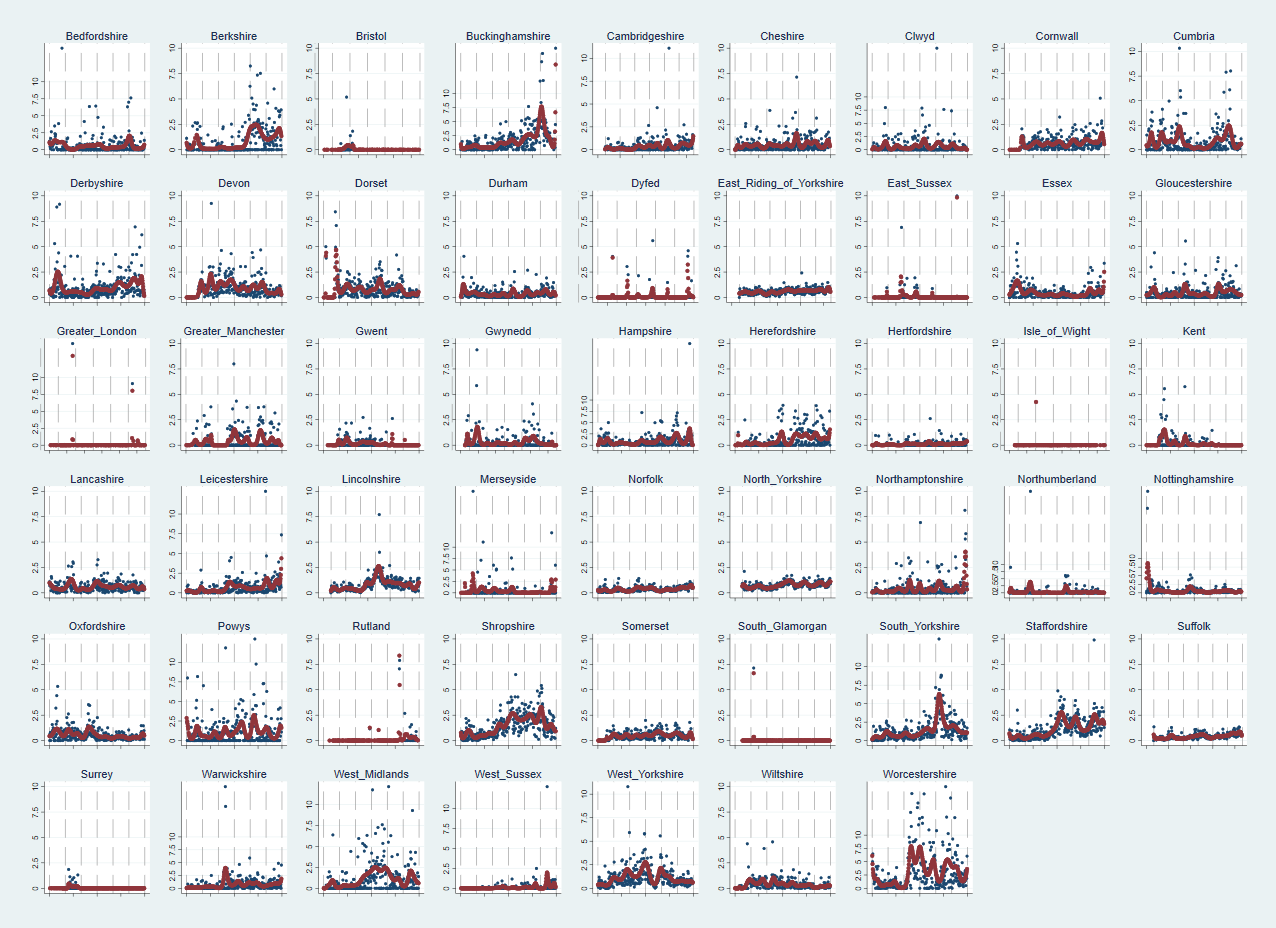

Supplement: Supplementary file 1 [file S0950268819002085sup001.zip › S0950268819002085sup001/Supplementary Figure S2 Tail biting counties.tif]

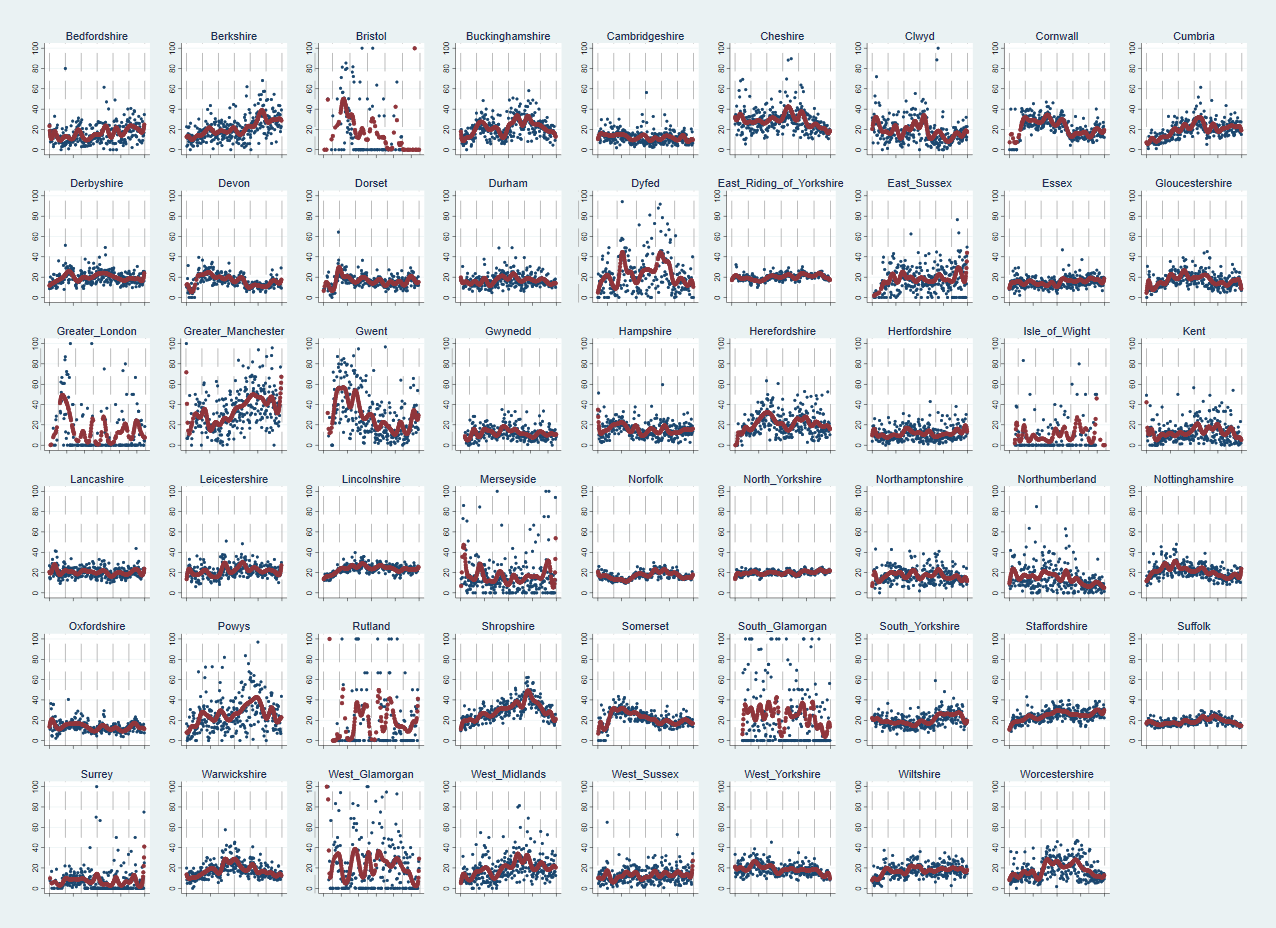

Supplement: Supplementary file 1 [file S0950268819002085sup001.zip › S0950268819002085sup001/Supplementary Figure S3 Respiratory counties.tif]
